# Cross-cultural validity of the five items *Mindful Attention Awareness Scale* (MAAS-5) in Peru and Mexico during the COVID-19 pandemic

**DOI:** 10.1186/s41155-022-00218-y

**Published:** 2022-05-17

**Authors:** Mario Reyes-Bossio, Emilio Lafferrnaderie Zapparigli, Tomás Caycho-Rodríguez, Carlos Carbajal-León, Luis Alberto Olavarria Castaman, Giovana Lourdes Hernandez Pino, Raymundo Calderon Sanchez, Sergio Barbosa-Granados

**Affiliations:** 1 Facultad de Psicología, Universidad Peruana de Ciencias Aplicadas, Lima, Perú; 2 Facultad de Ciencias de la Salud, Universidad Privada del Norte, Av. Alfredo Mendiola 6062, Los Olivos, Lima, Perú; 3 Facultad de Ciencias de la Salud, Universidad del Valle de México, Ciudad de México, México; 4 Universidad Cooperativa de Colombia, Medellín, Colombia

**Keywords:** Cross-cultural, Invariance, Mexico, Mindfulness, Peru

## Abstract

The Spanish version of the 5-item Mindful Attention Awareness Scale (MAAS-5) is a brief measure of the general tendency to be attentive and aware of experiences in the present moment during daily life. The MAAS-5 has been used in different countries; however, an assessment of its cross-cultural measurement invariance (MI) has not been conducted. Therefore, the study aimed to evaluate the cross-cultural measurement invariance of the MAAS-5 in university students from two countries: Peru and Mexico. A total of 1144 university students from Peru (*N* = 822) and Mexico (*N* = 322) responded online to the Spanish version of the 5-item Mindful Attention Awareness Scale (MAAS-5). A multigroup confirmatory factor analysis was performed. Measurement invariance tests the hypothesis that the model behind a set of scores is comparable between groups. The results showed that the unidimensional structure of the MAAS-5 is the same between Peruvian and Mexican university students. Therefore, it is suggested that university students from both countries conceptualize the mindfulness in a similar way. As a result, the MAAS-5 can be used to compare differences between countries. No significant differences were observed in the MAAS-5 score between Peruvian and Mexican university students. The present study contributes to a better understanding of the psychometric properties of the MAAS-5 by presenting MI results in two Latin American countries. Implications of the findings are discussed, which will facilitate a more solid and reliable use of the MAAS-5 in future cross-cultural studies.

## Introduction

The current COVID-19 pandemic is a severe stressor that has harmful effects on the mental health of the population worldwide (Xiang et al., 2020), generating fear about the risks the disease poses to one’s own and others’ health, the increased symptoms of post-traumatic stress disorder, anxiety, depression, and anger (Vindegaard & Benros, 2020; Xiong et al., 2020). These consequences affect different vulnerable groups such as medical staff (Lai et al., 2020) and people with mental disorders (Rohde et al., 2020), as well as the general population (Salari et al., 2020; Serafini et al., 2020). A review indicated that the prevalence of psychological problems during the pandemic was highest among COVID-19 patients, followed by health professionals and the general population (Krishnamoorthy et al., 2020). Another group considered vulnerable to mental health problems during the pandemic are university students (Huang et al., 2021). The COVID-19 pandemic has interrupted face-to-face education, generating a change to Internet-based learning, which with the effects of social isolation generates stress, increased symptoms of anxiety and depression, as well as greater isolation and uncertainty about the future of students (Gagliardi, 2020; González-García et al., 2021; Sun et al., 2021; Tang et al., 2020). However, health professionals in training not only face stress to maintain the continuity of their learning process but also, have to become part of the health response (Valdez-García et al., 2020). This is reflected culturally in several American countries, such as Mexico and Peru, which suffered difficulties in accessing the Internet, training, and learning of teachers and students for asynchronous classes, and problems adapting educational processes to exceptional contexts such as this one (Gagliardi, 2020). Despite all the inconveniences that university students may be involved, this scenario makes it necessary to identify protective factors that allow better mental health during the pandemic (Holmes et al., 2020). In addition, to allow them to have soft skills that allow them to develop efficiently in the various situations that arise (Urbina-Soto, 2019).

The term mindfulness is originated in the Buddhist tradition, then exported to Western countries, and then returned to the East in the form of therapeutic interventions and psychological measures (Karl et al., 2020). In the West, mindfulness is defined as “paying attention in a particular way, with purpose, in the present moment and without judgment” (Kabat-Zinn, 1994, p. 4). In addition, mindfulness can also be understood as a disposition or characteristic, referring to a stable tendency (whether natural or trained) towards conscious states in daily life (Brown & Ryan, 2003). Mindfulness-based interventions improve mental health by combining relaxation practices, meditation and elements of cognitive-behavioral therapy (Kabat-Zinn, 2011; Wielgosz et al., 2019). Specifically, mindfulness-based therapies are effective in reducing symptoms of depression, anxiety, and stress (Goldberg et al., 2019; Goyal et al., 2014; Hofmann et al., 2010; Khoury et al., 2013). Furthermore, this type of intervention has been shown to be effective not only in clinical populations, but also in healthy individuals (Khoury et al., 2015). Recently, research has suggested that mindfulness-based interventions mitigate the psychological impact of the COVID-19 pandemic (Bäuerle et al., 2020; Lim et al., 2021; Matiz et al., 2020). Mindfulness interventions delivered online have been shown to promote the mental health of college students during the COVID-19 pandemic by significantly decreasing levels of stress, depression, and anxiety (González-García et al., 2021). However, it must be recognized that the magnitude of the effect of mindfulness-based interventions and the methodological quality vary between studies (Sauer et al., 2013). This has led to the existence of a debate about the methodological aspects in studies on mindfulness, which includes its evaluation (Bergomi et al., 2013; Davidson & Kaszniak, 2015; Sauer et al., 2013). Assuming that interventions based on mindfulness have significant effects would depend on the evidence of reliability and validity of the instruments to measure a complex construct such as this one (Goodman et al., 2017). Therefore, having measures that examine the effects of mindfulness training are important to test the theoretical evidence on the topic (Abujaradeh et al., 2020). Likewise, the measurement of mindfulness is important to better understand its relationships with health and well-being (Baer, 2019). Mindfulness measurement is important for in-depth evaluation of the efficacy of interventions that seek to teach these skills (Baer et al., 2004). Similarly, measures of mindfulness are useful for understanding its nature and the mechanisms by which mindfulness-based interventions generate beneficial effects. (Brown & Ryan, 2004; Dimidjian & Linehan, 2003). In this sense, rigorous studies of treatment outcomes are necessary to identify the role of mindfulness. In this scenario, mindfulness measures can be very helpful.

Currently, there are different measures of mindfulness, which are based on different operational definitions (Baer, 2019; Iani et al., 2020; Sauer et al., 2013). The use of self-report psychometric scales is the most widely used approach to measure mindfulness due to its fast application, known methodology, and the presence of empirical support. (Bohlmeijer et al., 2010; Chiesa et al., 2011). On the other hand, there are also criticisms of this methodology that can limit the findings, such as the lack of a common definition of mindfulness and the presence of an introspective self-perception, typical of self-report measures, which can generate bias, due to a superficial understanding of mindfulness (Grossman, 2008). However, these criticisms are not exclusive to studies on mindfulness, but rather it is a problem that limits the usefulness of self-report instruments in general in different research topics (Sauer et al., 2013). In addition, there are instruments that operationalize mindfulness as a one-dimensional construct, while others consider a multifactorial structure (Sauer et al., 2013).

One of the most popular self-reporting measures is the *Mindful Attention Awareness Scale* (MAAS; Brown & Ryan, 2003) which measures the general tendency to be attentive and aware of experiences in the present moment during daily life and that can be experienced in different situations. The MAAS has several advantages compared to other mindfulness scales, such as its brevity, unidimensional structure, the possibility of being applied to both clinical and non-clinical populations, without the need for previous experience in meditation, and availability in numerous languages, rather than any other measure of mindfulness (González-Blanch et al., 2022). The MAAS has been translated and adapted to different languages, such as Portuguese (Barros et al., 2015), Spanish (Johnson et al., 2014; Montes et al., 2014), Chinese (Deng et al., 2012), and Italian (Veneziani & Voci, 2015) among others. In addition, its psychometric properties have been extensively evaluated from Classical Test Theory and Item Response Theory (Van Dam et al., 2010). However, the presence of some MAAS items has also been questioned (Caycho-Rodríguez et al., 2019, b), which do not allow discerning different levels of *mindfulness* (Chiesi et al., 2017; Van Dam et al., 2010). The above has generated that, along with the original 15-item version of the MAAS, there are shorter versions of 10 items (Goh et al., 2017), 13 items (Medvedev et al., 2016), 6 items (Black et al., 2012), and 5 items (Van Dam et al., 2010).

The 5-item version of the MAAS (MAAS-5) is composed of ítems 7, 8, 9, 10, and 14 of the original version and presents adequate discrimination between different levels of mindfulness, as well as information similar to the 15-item version (Van Dam et al., 2010). Other studies, in different languages, also suggest that the MAAS-5 is consistent with the unidimensional conceptual basis of the original MAAS, has adequate reliability, and is gender invariant (Caycho-Rodríguez et al., 2021; Caycho-Rodríguez, García Cadena, et al., 2019; Goh et al., 2017; Osman et al., 2016; Smith et al., 2017).

The MAAS-5 has been translated into Spanish (Caycho-Rodríguez, Ventura-León, et al., 2019) and its psychometric properties have been evaluated in both university students (Caycho-Rodríguez, García Cadena, et al., 2019) and Peruvian older adults (Caycho-Rodríguez et al., 2021). However, the psychometric equivalence of the MAAS-5 among other Spanish-speaking countries has not been evaluated. Recently, it has become important to assess how valid the instruments are for measuring mindfulness across different cultures (Karl et al., 2020). This can be evaluated on the basis of measurement invariance (MI) across different cultural groups (Milfont & Fischer, 2010). Cross-cultural MI refers to the comparability of a measure across cultures. In this way, a measure has MI if two or more groups respond to the instrument in the same way (Cheung & Rensvold, 2002; Milfont & Fischer, 2010). MI can be empirically assessed at three levels: configural, metric, and scalar invariance (van de Vijver & Leung, 2011). Configural invariance refers to the fact that all items can be used to measure the same construct across cultures and, moreover, these items conform to the same dimensional structure across groups (Fischer & Fontaine, 2011). On the other hand, metric invariance implies that all items have similar factor loadings in all groups. Finally, scalar invariance suggests that the intercepts of the items are identical in all groups. This would indicate that people with the same level of mindfulness would respond similarly to each item across cultures and, moreover, their responses would not be affected or vary due to possible response patterns, such as acquiescence, reference group effect, or social desirability (van de Vijver & Leung, 2011).

Therefore, it is necessary to have measures that can be used to assess changes in mindfulness in different cultures (Raphiphatthana et al., 2019). Cross-cultural studies have shown that people with different cultural backgrounds differ in the conceptualization and experiences of mindfulness (Ivtzan et al., 2018). For example, it has been reported that U.S. students had greater psychological needs and were more aware than Turkish students (Özyeşil, 2012). It has also been indicated that American students tend to be more accepting of events that occurred around them; meanwhile, Thai students were better able to concentrate with full attention (Christopher et al., 2009). There are currently studies that have suggested that different instruments for measuring mindfulness, such as the Child and Adolescent Mindfulness Measure or el Five-Facet Mindfulness Questionnaire (García-Rubio et al., 2019; Karl et al., 2020) can be used successfully in different cultures. However, there is no study that evaluates the cross-cultural MI of MAAS-5. In this sense, the current research aimed to evaluate the cross-cultural MI of the MAAS-5 among university students in Peru and Mexico. In Peru, studies have suggested that university students with higher levels of mindfulness have shown fewer symptoms of anxiety and depression, decreased anger, as well as improved psychological well-being, life satisfaction, and compassion. (Caycho-Rodríguez, Cabrera-Orosco, et al., 2020; Caycho-Rodríguez, García Cadena, et al., 2019; Caycho-Rodríguez, Vilca, et al., 2020). In Mexico, university students with a higher level of mindfulness, have a better perception of their quality of life, are more satisfied with their life and experiment less stressful academic situations (Meda Lara et al., 2015). There are no reported studies on mindfulness in Peru and Mexico during the COVID-19 pandemic. MAAS-5 is expected to present MI between Peru and Mexico. The previous hypothesis is based on the fact that both countries share influences and cultural characteristics, which would generate a similar way of learning and practicing mindfulness (Demarzo et al., 2015). In addition, it is suggested that in Latin American countries where the family plays an important role, such as Peru and Mexico, mindfulness can be well received due to the need to pay attention and be aware of one’s own emotions (García-Campayo et al., 2017). Therefore, the evaluation of MI will provide information on the comparability of the mindfulness construct between the two countries and the type of comparisons that can be made. The lack of cross-cultural MI would indicate that the MAAS-5 is not a suitable measure for cross-cultural research, so further research would be needed to establish a conceptualization of mindfulness that can be valid across cultures.

## Method

### Participants

A total of 1144 university students from Peru (*N* = 822) and Mexico (*N* = 322) participated. The minimum number of participants in each country was made with Soper’s software (2021). For this, 5 observed variables (the five items of the MAAS-5), 1 latent variable (mindfulness), an anticipated effect size (λ = 0.3), probability (α = 0.05) and statistical power (1-β= 0.95). Thus, the final number of participants was much higher than the suggested minimum number of 100 participants. Furthermore, as proposed by Hoe (2008), any study with a sample of more than 200 participants can provide sufficient statistical power to carry out a data analysis. In the Mexican sample, 19.25% were men and 80.75% were women, with an average age of 20.82 years (SD = 3.93). In Peru, 148 men (18%) and 674 women (82%) participated, with an average age of 20.77 years. (SD = 2.23). Table 1 presents in more detail the sociodemographic characteristics in both countries. In both Peru and Mexico, participants were selected by non-probabilistic sampling, with the inclusion criteria being to be enrolled in a private university in Peru and Mexico at the time of the study.

**Table 1:** Sociodemographic characteristics of the samples from Peru and Mexico

	Mexico	Peru
	*n*(%)	*n*(%)
Sex		
Male	62 (19.25)	148 (18)
Female	260 (80.75)	674 (82)
The place where the quarantine is passing has		
Less than 70 m^2^	30 (9.3)	70 (8.5)
Between 70m^2^ and 90 m^2^	72 (22.4)	177 (21.5)
Between 90 m^2^ and 120 m^2^	108 (33.5)	304 (37)
More than 120 m^2^	112 (34.8)	271 (33)
The place where you spend the quarantine has a garden or outdoor terrace		
Yes	190 (59)	619 (75.3)
No	132 (41)	203 (24.7)
Number of hours you do physical activity during quarantine		
Less than 5 h	181 (56.2)	384 (46.7)
5–7 h	37 (11.5)	182 (22.1)
8–10 h	10 (3.1)	57 (6.9)
11–13 h	6 (1.9)	35 (4.3)
14–16 h	3 (0.9)	28 (3.4)
More than 16 h	7 (2.2)	31 (3.8)
Does not apply	78 (24.2)	105 (12.8)
Have you been able to organize yourself to study?		
Never	0 (0)	37 (4.5)
Rarely	31 (9.6)	176 (21.4)
Sometimes	98 (30.4)	287 (34.9)
Most of the time	140 (43.5)	240 (29.2)
Forever	53 (16.5)	82 (10)
Has your physical activity decreased during quarantine?		
Never	29 (9)	111 (13.5)
Rarely	50 (15.5)	142 (17.3)
Sometimes	60 (18.6)	191 (23.2)
Most of the time	101 (31.4)	227 (27.6)
Forever	82 (25.5)	151 (18.4)
How many hours approximately do you sleep at night?		
0–4 h	44 (13.7)	67 (8.2)
4–8 h	190 (59)	515 (62.7)
8–12 h	86 (26.7)	225 (27.4)
More than 12 h	2 (0.6)	15 (1.8)
Have you gained weight?		
Between 1 and 2 kg	131 (40.7)	333 (40.5)
Between 3 and 4 kg	61 (18.9)	131 (15.9)
More than 4 kg	21 (6.5)	42 (5.1)
Nothing	109 (33.9)	316 (38.4)
Have you come to consult with the psychologist at this time?		
0 times	234 (72.7)	604 (73.5)
Between 1 and 3 times	53 (16.5)	137 (16.7)
Between 4 and 5 times	8 (2.5)	34 (4.1)
Between 6 and 8 times	10 (3.1)	21(2.6)
More than 8 and times	17 (5.3)	26 (3.2)

### Instruments

*Mindful Attention Awareness Scale of five items* (MAAS-5; Van Dam et al., 2010). The MAAS-5 is a short version of the original 15-item MAAS (Brown & Ryan, 2003). In the present study, the translated and validated version in Spanish for university students was used (Caycho-Rodríguez, García Cadena, et al., 2019; Caycho-Rodríguez, Ventura-León, et al., 2019). The five items of the MAAS-5 have six Likert-type response options (6 = almost never to 1 = almost always) and measure individuals’ ability to pay attention during various tasks or, conversely, behave automatically without paying sufficient attention. No previous meditation experience is necessary to answer the MAAS-5. Examples of the items are “I perform my activities quickly, without being very attentive to what I am doing”, “I do work automatically, without noticing what I am doing” (see all items in Table 2). Higher MAAS-5 scores indicate a higher level of mindfulness.

**Table 2:** Mean (M), standard deviation (SD), skewness (g1), kurtosis (g2), standardized factor loads (λ), and test item correlation (*r*_it_)

	Total sample					Sample Mexico					Sample Peru				
	M(SD)	g1	g2	λ	*r*_it_	M(SD)	g1	g2	λ	*r*_it_	M(SD)	g1	g2	λ	*r*_it_
Ítem 1	3.61(1.58)	.07	− .99	.76	.67	3.47(1.57)	.15	− .97	.78	.68	3.70(1.59)	.03	− 1.05	.75	.65
Ítem 2	3.68(1.46)	.08	− .88	.83	.74	3.62(1.42)	.07	− .79	.81	.73	3.71(1.48)	.07	− .93	.84	.75
Ítem 3	3.79(1.42)	− .01	− .85	.65	.56	3.65(1.36)	.04	− .65	.66	.58	3.87(1.44)	− .02	− .95	.64	.56
Ítem 4	3.93(1.53)	− .19	− .99	.90	.79	3.86(1.54)	− .21	− .99	.88	.78	3.97(1.53)	− .19	− 1.01	.91	.80
Ítem 5	3.62(1.54)	.06	− .99	.81	.71	3.60(1.53)	− .01	− 96	.79	.69	3.61(1.55)	.11	− 1.03	.80	.71

### Procedure

In both Peru and Mexico, participants completed an online survey, developed on a Google form. Participants were also asked to give their informed consent online before starting the survey, and no one was financially compensated for their participation. In this sense, participation was voluntary, anonymous and followed the ethical guidelines for conducting research at each of the participating universities. The research was approved by the Research Subcommittee of the Faculty of Health Sciences and Psychology of the Peruvian University of Applied Sciences (FCS/CEI 165-06-20). Data were collected between June 23 and August 21, 2020. During this period, in Peru, there was an increase from 268,602 to 585,236 diagnosed cases of COVID-19, as well as from 8761 to 27,453 deaths from the disease. In Mexico, more than 462,690 confirmed cases and 50,517 deaths from COVID-19 were recorded during the data collection period.

### Data analysis

First, descriptive statistics were calculated for the MAAS-5 items (averages, standard deviations, asymmetry, and kurtosis). The internal structure was evaluated by means of a confirmatory factor analysis (CFA), using the estimator Diagonally Weighted Least Squares with Mean and Variance corrected (WLSMV) due to the ordinal nature of the items (Brown, 2015). The model evaluated in the CFA was the one suggested in the Spanish scientific literature (Caycho-Rodríguez et al., 2021; Caycho-Rodríguez, Ventura-León, et al., 2019). The model fit was evaluated on the basis of different goodness-of-fit indices: Confirmatory Fit Index (CFI) and Tucker-Lewis Index (TLI), both with appropriate values ≥ .90; the standardized root mean square residuals (SRMR) and the root mean square error of approximation (RMSEA), with a confidence interval of 90% and where values < .08 are suitable (Hu & Bentler, 1999). Reliability was assessed by calculating two internal consistency coefficients: Cronbach’s alpha (α) and McDonald’s Omega (ω). Both are expected to have values above .80 to be considered adequate reliability (Kelley & Pornprasertmanit, 2016; McDonald, 1999).

After confirming the factor structure of the MAAS-5, MI was evaluated with respect to country. Because the sample sizes of the groups were very different, a subsampling with 100 repetitions suggested in the literature was performed (Yoon & Lai, 2018). In this sense, a subset of the largest sample is randomly selected 100 times, where each subsample is used for MI analysis together with the sample from the smallest group. In this sense, the MI is performed 100 times for the different subsamples of the larger group, while the same sample is used in the smaller group for all 100 analyses. Thus, the fit indices are calculated for each analysis, calculating the average value of each fit index in the 100 replicates. A sequence of increasingly restrictive hierarchical variance models was evaluated. First, configurational invariance was tested (considered as the reference model), followed by metric invariance (where factor loadings were equal between groups), scalar invariance (where factor loadings and intercepts were equal), and strict invariance (equality of factor loadings, intercepts, and residuals). The change in CFI (ΔCFI) was used as the main criteria to compare models with more restrictions to models with fewer restrictions, where a ΔCFI of < .01 provides evidence of MI (Chen, 2007; Putnick & Bornstein, 2016). The ΔCFI was preferred over *χ*2 comparisons as the MI criteria, since the former is not sensitive to sample size (Putnick & Bornstein, 2016; Widaman & Reise, 1997). The differences between the latent means of the groups of university students from both countries were estimated. To compare latent means, the mean of the latent factor was set to zero in the reference group (Peruvian group) and was left to vary freely in the comparison group. Comparison of latent means was performed using the critical ratio (CR) index, where CR values ≥ ± 1.96 indicate statistically significant differences in means at *p* ≤ .05. Positive CR values suggest that the comparison group has larger latent means than the reference group (Tsaousis & Kazi, 2013). A measure of effect size, such as Cohen’s *d* (*d*), was used to measure the magnitude of the differences, where a *d* = .20, *d* = .50, and *d* = .80 indicate small, medium, and large effect sizes, respectively (Cohen, 1992).

Statistical analysis was performed with the RStudio environment (RStudio Team, 2018) for R (R Core Team, 2019), using the “lavaan” package (Rosseel, 2012) for CFA, and the “semTools” package (Jorgensen et al., 2018) to evaluate MI.

## Results

### Descriptive analysis of the MAAS-5 items

Average, standard deviation, asymmetry, and kurtosis were calculated for all MAAS-5 items. Table 2 shows that item 4 (“I do work automatically, without realizing what I am doing”) has the highest average in the total sample; while, in the total and Peruvian sample, item 1 (“It seems as if I am working on “automatic pilot”, without being very aware of what I am doing”) had the lowest average. In the Mexican sample, item 5 (“I realize that I do things without paying attention”) has the lowest mean. As for the asymmetry (g1) and kurtosis (g2) indices, it can be observed that all the items present adequate indices (g1 and g2 < ± 2), according to the criteria of Finney and DiStefano (2006).

### Factor analysis and reliability

In relation to the MAAS-5 single factor model fit, the chi-square test was significant (*p* < .001), in the total sample, Peruvian sample and Mexican sample, indicating possible discrepancies. However, the chi-square test is affected by a large sample size (). In this sense, given that CFA is a technique that makes use of relatively large samples, it is not uncommon to have a statistically significant chi-square. As such, the CFI, TLI, RMSEA, and SRMR values showed that the MAAS-5 single factor model fit the data well in all samples (total, Peruvian, and Mexican) (see Table 3). Furthermore, all factor loadings were statistically significant (*p* < .001). As shown in Table 2, the standardized factor loadings of all items, in all samples, were greater than .60 (the loadings ranged from .64 to .91), as recommended by Pituch and Stevens (2016).

**Table 3:** Fit indices and invariance models

	*χ*^2^	df	*p*	SRMR	TLI	CFI	RMSEA	ΔCFI	ΔRMSEA	ΔSRMR
Unidimensional model										
Total sample	27.09	5	.000	.025	.997	.999	.062	–	–	
Peru sample	20.61	5	.000	.026	.998	.999	.061	–	–	
Mexico sample	14.43	5	.013	.028	.997	.998	.064	–	–	
By country										
Configural	107.35	10	.000	.029	.970	.990	.131	–	–	
Metric	147.65	25	.000	.029	.990	.987	.093	− .003	− .038	.000
Scalar	127.86	29	.000	.029	.993	.990	.077	.003	− .015	.000
Strict	169.34	33	.000	.030	.991	.986	.085	− .004	.008	.000

*χ2* chi-square, *df* degrees of freedom, *SRMR* standardized root mean square residual, *TLI* Tucker-Lewis Index, *CFI* Comparative Fit Index, *RMSEA* root mean square error of approximation, *ΔRMSEA* change in root mean square error of approximation, *ΔCFI* change in comparative fix index, *ΔSRMR* change in standardized root mean square residual

Cronbach’s alpha (α) and McDonald’s Omega (ω) of the total sample (α = .87; ω = .90), Peruvian sample (α = .88; ω = .89), and Mexican sample (α = .89; ω = .90), indicate a very good internal consistency of the MAAS-5 in Spanish. Furthermore, as shown in Table 2, the corrected item-total correlations were positive and with values between .56 and .80. The results demonstrate that the MAAS-5 in Spanish is a unidimensional and reliable measure.

### Cross-cultural invariance and comparison of latent means

The goodness of fit indices of the different MI models between the Peruvian and Mexican groups, as well as the variations in CFI and RMSEA are presented in Table 3. The MAAS-5 single factor model has been shown to be strictly invariant between Peruvian and Mexican university students (ΔCFI < .01). Therefore, comparisons can be made between these groups.

The comparison of means allows an estimation of the difference of means in the latent variable (in this case, mindfulness) between the groups (university students from Peru and Mexico). The results indicate that there is no significant difference between the latent means of mindfulness according to the country (CR = 1.21). No significant magnitude of difference was observed at the practical level (*d* = .133, 95% CI .010–.243).

## Discussion

To our knowledge, this is the first study that aimed to evaluate the MI of the MAAS-5 in two Latin American countries: Peru and Mexico. The evaluation of the internal structure of the MAAS-5 in Spanish provided support for a unidimensional factor model, where factor loadings were generally high, ranging from .64 to .91 for the total sample, the Peruvian sample, and the Mexican sample. The presence of high factor loadings indicates that most of the variance of mindfulness is explained by the items included in the MAAS-5, which supports the reliability of the instrument. Likewise, the presence of the unidimensional model is consistent with the theoretical and empirical development of the scale (Brown & Ryan, 2003) and with validation studies in Spanish carried out in university students (Caycho-Rodríguez, Ventura-León, et al., 2019) and older Peruvian adults (Caycho-Rodríguez et al., 2021). In this sense, having a one-dimensional structure can be used as a basis for evaluating mindfulness, as a preliminary step to expanding the concept in several dimensions, taking into account that a one-dimensional structure is easier to study and interpret, compared to one multidimensional structure (Poorebrahim et al., 2021). The results of the present study also demonstrated that the MAAS-5 has high reliability, and these findings are in line with previous studies based on samples from different countries (Caycho-Rodríguez et al., 2021; Caycho-Rodríguez, García Cadena, et al., 2019; Goh et al., 2017; Osman et al., 2016; Smith et al., 2017).

The results of the multigroup CFA supported the presence of a single MAAS-5 factor in each of the two countries. Specifically, the configurational model was supported, confirming that a single latent factor is similar in both countries. This suggests that university students in Peru and Mexico conceptualize mindfulness in a similar way. Metric invariance was also tested, where no differences in factor loadings between countries were observed. The presence of metric invariance indicates that the five MAAS-5 items measure mindfulness behaviors in the same way in the two national samples. Consequently, the behaviors indicated in the five items (e.g., automatic work, inattention) appear to be equally indicative of the single MAAS-5 dimension among Peruvians and Mexicans (Oort, 2005). The results also indicate the presence of scalar invariance that allows comparisons of latent means. Finally, strict invariance would indicate that the systematic measurement error associated with belonging to a given group (Peruvian or Mexican university students) is equivalent between groups. Providing evidence that mean comparisons based on observed variables would reflect differences in latent traits, and that they are not influenced by unknown errors, is important for cross-cultural mindfulness research. Tests of invariance across countries also provide evidence of validity and reliability for the MAAS-5. In this way, the findings suggest that the MAAS-5 can be reliably applied to university students from both countries.

Testing for scalar invariance, the findings revealed that the average MAAS-5 scores in Spanish did not differ between the Peruvian and Mexican groups of university students. This could be explained, in part, by the precise unidimensional conceptualization of mindfulness as an attentional approach. In addition, Latin countries, such as Peru and Mexico, share cultural influences and standards, which give rise to a similar way of learning and practicing mindfulness, different from what occurs in non-Latin countries (Demarzo et al., 2015). Similarly, mindfulness is well received by people in these countries because the family plays a crucial role in Latin American countries, which requires awareness of one’s own emotions (García-Campayo et al., 2017). Likewise, informal mindfulness practice within the family environment is frequently reported by people from Latin American countries (Garcia-Campayo & Demarzo, 2014).

Despite the results, some limitations of the study should be considered. First, only two Spanish-speaking Latin American countries were considered in the study. Other studies would benefit from including more countries and applying different translations of the scale (such as English and Portuguese). Second, the generalizability of the findings is limited due to the use of convenience sampling. Third, only students from private universities were included. Therefore, it would be important to replicate the study in students from public universities. Fourth, the sample sizes were unequal between the groups and a greater number of women than men is observed (this is a product of the type of sampling used). Although this difference in sample size could affect the results obtained by the multigroup factor analysis (Brown, 2015), the subsampling approach recommended by Yoon and Lai (2018), and used in this study, attempted to mitigate this problem. In addition, the use of ΔCFI also contributed to mitigate this problem, since it is independent of model complexity and sample size (Cheung & Rensvold, 2002). As such, future studies could adjust the sample sizes. Fifth, the cross-sectional design did not allow to evaluate test-retest reliability or longitudinal MI. Sixth, evidence of convergent, discriminant and incremental validity of the Spanish version of the MAAS-5 was not evaluated in this study. Seventh, because the objective of the study was specific (to assess MI between different countries), MI between different sexes was not evaluated. Previous reviews have reported differences in the effects of mindfulness-based interventions by gender (Katz & Toner, 2013); however, other studies have found no such differences (Sedlmeier et al., 2012). In this sense, it is important that future studies consider gender differences in the experience of mindfulness in order to have a better understanding of the problem.

## Conclusions

Despite the limitations, the present study contributes to a better understanding of the psychometric properties of the MAAS-5 by presenting MI results in two Latin American countries. Therefore, it has been demonstrated that the MAAS-5 can be useful to meaningfully compare scores between different countries. Without MI verification it cannot be assumed that the comparative results are valid (Chen, 2008). In addition, it is important to note that this study was applied in countries other than the USA and other European countries where the scale has been applied, which allows us to extend the findings to multicultural contexts. The findings on the psychometric properties and cross-cultural utility of the MAAS-5 contribute to understanding the theoretical foundations of mindfulness as a unidimensional construct and provide researchers with evidence for selecting appropriate indicators to assess this construct. The MAAS-5 can facilitate the evaluation of the effects mindfulness-based intervention during the current COVID-19 pandemic in the university setting. This is even more important if we consider that the practice of mindfulness is a low-cost intervention that mitigates the psychological impact produced by the COVID-19 pandemic on university students (González-García et al., 2021). The COVID-19 pandemic has demonstrated the presence of constant change, where mindfulness can provide a useful way to adapt to these constant changes (Behan, 2020). Finally, it is hoped that these results will motivate other researchers to evaluate the MAAS-5 MI before comparing mindfulness across different countries.

## Data Availability

All data related to this study are available from the authors upon request. The data are not yet publicly available because the project group is still processing it.
